# Glass ionomer cements with milled, dry chlorhexidine hexametaphosphate filler particles to provide long-term antimicrobial properties with recharge capacity

**DOI:** 10.1016/j.dental.2018.09.003

**Published:** 2018-12

**Authors:** Candice A. Bellis, Owen Addison, Angela H. Nobbs, Peter F. Duckworth, James A. Holder, Michele E. Barbour

**Affiliations:** a Oral Nanoscience, Bristol Dental School, University of Bristol, UK; b School of Dentistry, University of Birmingham, UK; c Oral Microbiology, Bristol Dental School, University of Bristol, UK; d ACCIS, University of Bristol, UK; e Kemdent, Purton, UK; f Pertinax Pharma Ltd., Bristol, UK

**Keywords:** BFS, biaxial flexural strength, CHX, chlorhexidine, CS, compressive strength, DTS, diametral tensile strength, GIC, glass ionomer cement, HMP, hexametaphosphate, Glass ionomer cement, Restorative materials, Caries, Antimicrobials, Chlorhexidine

## Abstract

**Objective:**

Glass ionomer cements (GICs) are a versatile material, offering the opportunity for ion exchange with the oral environment. The aim of this study was to develop a GIC that delivers a controlled, rechargeable dose of chlorhexidine (CHX) over an extended period without compromising mechanical properties.

**Methods:**

GICs were supplemented with finely milled particles of chlorhexidine hexametaphosphate (CHX-HMP). CHX release into artificial saliva was measured over 660 days, and recharge with CHX and CHX-HMP was investigated. Mechanical properties were investigated, and an agar diffusion test was carried out to assess antimicrobial properties using *Streptococcus mutans* and *Scardovia wiggsiae*.

**Results:**

Dose-dependent CHX release was observed, and this was ongoing at 660 days. Compared with related studies of GICs containing CHX-HMP, the fine, dry particles resulted in fewer adverse effects on mechanical properties, including tensile, compressive and biaxial flexural strength, with 1% CHX-HMP GICs indistinguishable from control specimens. The GICs could be recharged with CHX using both a conventional CHX digluconate solution comparable to commercial mouthrinses, and a suspension of CHX-HMP of equivalent concentration. Recharging with CHX digluconate increased subsequent CHX release by 50% compared with no recharge, and recharging with CHX-HMP increased subsequent CHX release by 100% compared with no recharge. The GICs inhibited growth of *St. mutans* and *Sc. wiggsiae* in a simple agar diffusion model.

**Significance:**

These materials, which provide sustained CHX release over clinically relevant timescales, may find application as a restorative material intended to inhibit secondary caries as well as in temporary restorations and fissure sealants.

## Highlights

• Milled, dry chlorhexidine hexametaphosphate (CHX-HMP) was incorporated into a GIC.
• CHX release was dose-dependent and sustained for at least 660 days.
• GICs could be recharged with CHX and CHX-HMP.
• 1% CHX-HMP did not affect compressive, tensile or biaxial flexural strength.
• CHX-HMP GICs inhibited growth of cariogenic microorganisms in an agar diffusion model.

## 1 Introduction

Glass ionomer cements (GICs) are a mainstay of modern dentistry. Their uses include temporary and permanent restorations, lining and luting materials, fissure sealants and in atraumatic restorative therapy. Although GICs exhibit fluoride release, and antimicrobial effects of this can be demonstrated *in vitro*, the data in support of an anticariogenic effect *in vivo* is inconclusive [1], [2], [3]. Nevertheless, a bioactive material that participates in ion exchange with its local environment offers an adaptable vehicle for delivery of other useful molecules to the locale of a restoration.

Chlorhexidine (CHX) is a broad-spectrum antimicrobial in widespread use as a topical agent. It owes its antimicrobial properties to membrane disruption and is efficacious against a wide range of microbes including those implicated in caries. CHX in commercial products is typically present as the CHX digluconate salt which is highly soluble and thus has poor substantivity, usually providing only short term antimicrobial function before it is depleted. CHX concentrations in saliva fall rapidly after an oral rinse, with 90% of the initially retained CHX cleared from the mouth within 6 h and 98% cleared within 12 h [4]. One product used for treatment of periodontal disease provides sustained CHX release, but 80% of the CHX is released within the first 2 days, with a much lower release over the following 3–4 weeks [5].

Owing to the useful antimicrobial functionality of CHX, coupled with its low cost and well-understood pharmacological properties, there have been attempts to incorporate CHX into dental materials, including GICs. GICs supplemented with CHX diacetate and CHX digluconate inhibited growth of oral microbes *Streptococcus mutans* and *Lactobacillus acidophilus*, but the antimicrobial effects were sustained for only 40–90 days, with no bactericidal effect observed after this time [6]. In another study where GIC was doped with CHX digluconate and antimicrobial efficacy again assessed using *St. mutans* and *L. acidophilus*, there was an exponential decay of the zone of inhibition over the first 30 days [7]. These short-term antimicrobial effects may be explained by the release profiles of soluble CHX from GICs doped with these conventional salts, which show a high initial release which rapidly diminishes leaving little or no sustained release [8]. A recent report of core–shell microparticles containing CHX chloride salts reveals potential for graduate release of CHX, although this study only probed CHX release to a maximum of 430 h (18 days) [9]. Supplementation with these CHX salts has also been found to be associated with a negative impact on mechanical properties [6], [10], [11], [12]. More recently, some attempts have been made to incorporate CHX when loaded into particles that may facilitate gradual rather than rapid release, and there has been some success in this, but still the CHX release was heavily front loaded and decayed rapidly after the first 1–3 days [13].

Although *in vivo* studies of these experimental materials are rare, there are a few small studies of note. Supplementation of a resin-modified GIC with 1.25% CHX digluconate resulted in elimination of *St. mutans* populations following indirect pulp treatment *in vivo* [14], and incorporation of CHX digluconate into a GIC reduced microbial counts in cavities in first primary molars in 6–9 year olds, although the CHX digluconate GICs also exhibited a higher failure rate than the controls [15]; similar results were observed in a study of CHX digluconate doped GICs in children receiving atraumatic restorative therapy, where the CHX GICs resulted in reduced *St. mutans* counts at 7 days but not 3 or 12 months after placement [16]. These tentative findings highlight the potential benefit of CHX-functionalised restorative materials as well as the challenges, in that the other properties of the GIC are often compromised by the new component.

We have recently reported a novel salt of CHX: CHX-hexametaphosphate (CHX-HMP) [17]. CHX-HMP has a much lower solubility than CHX digluconate or CHX diacetate, and when in or on biomaterials, can confer a sustained release of CHX [18]. We have described a pilot study demonstrating the use of manually ground CHX-HMP as a filler for GICs [19], and another where the CHX-HMP was incorporated into a GIC as a viscous paste formulation [20]. In the first study [19], large clusters of CHX-HMP particles were used, and this had adverse effects on the mechanical properties; furthermore, another limitation of that study was the short time over which CHX release from the prototype cements was recorded, and the large particle size would have had a detrimental effect on this CHX release. In the second report [20], the incorporation of the particles in a wet aqueous paste resulted in deterioration of the mechanical properties when the substitution of CHX-HMP was at 0.34% by mass or greater, although the CHX release was improved on the earlier approach, being sustained for at least 14 months. The aim of the study described here was to investigate the use of CHX-HMP particles as GIC fillers but incorporating a mechanical milling process resulting in much smaller CHX-HMP filler particles. The hypothesis was that the smaller particle size and thus larger specific surface area would create a more sustained CHX release coupled with fewer adverse effects on mechanical properties. Furthermore, given that any antimicrobial incorporated into a biomaterial has a finite duration of release, the capacity of these materials to be “recharged” with CHX, in analogy to the well-known fluoride recharge phenomenon, was investigated. A simple microbiological test was incorporated into the study to determine whether CHX release from the prototype GICs had an effect on two cariogenic bacteria in an agar diffusion test. The conventional and well-characterised *St. mutans* was selected for the test, as well as *Scardovia wiggsiae*, a recently recognised anaerobic pathogen associated with aggressive childhood caries [21], [22].

## 2 Methods

### 2.1 Preparation of CHX-HMP filler particles

100 mL 10 mM solutions of each of CHX digluconate and sodium HMP (Sigma Aldrich, Gillingham, UK) were prepared in deionised water and were combined in a glass beaker under ambient laboratory conditions. The resulting aqueous suspension of CHX-HMP was stirred vigorously for approximately 1 min, then 30 mL 1 M potassium chloride was added and stirred for a further minute. The suspension was stored without disturbing for 24 h, allowing the precipitate to sediment at the base of the container. The supernatant was gently discarded leaving a concentrated suspension of the precipitate, which was then centrifuged at 4760 g for 30 min. The supernatant was again discarded and the pellet dried at ambient temperature under air extraction for 24 h, then removed from the centrifuge tubes. 100 g total mass of dried CHX-HMP pellets prepared in this way was placed into a cylindrical ceramic mill of 80 mm diameter and 70 mm height containing ceramic balls of sizes 12.5–20.5 mm (Pascal Engineering Company Ltd., Crawley, UK), and this was placed on a rolling platform for 4 h. The mill was opened every hour and particles dislodged from the internal walls. The resulting powder was passed through a coarse sieve and examined using scanning electron microscope (SEM). The specific surface area was measured by nitrogen adsorption following outgassing for 48 h at 30 °C under vacuum. Elemental microanalysis (CHNP) was used to confirm the composition of the precipitate; CHX-HMP was prepared as described above and analysed for C, H, N and P (Microanalytical Laboratory, School of Chemistry, University of Bristol).

### 2.2 Preparation of specimens (elution, DTS, CS)

Diamond Carve™ GIC (Kemdent, Purton, UK) was used as the base material to create experimental cements. This commercial GIC comprises a powder consisting of alumina-silica based glass filler particles which also contain calcium fluoride and other minor salt components and freeze-dried poly(vinyl)phosphonic acid, and a liquid, which contains polyacrylic and tartaric acids.

For the prototype GICs, CHX-HMP particles were substituted for the glass component in the GIC powder at 0, 1, 2, 5 and 10% by mass. All dopings were tested for each parameter below, unless otherwise specified. The CHX-HMP particles were mixed with the GIC powder in the ball mill for 10 min as described above to ensure homogeneous incorporation of the components. This powder was then mixed at a 4:1 ratio by mass with the GIC liquid according to the manufacturers’ instructions. Mixing was completed in 40–50 s and packing into the moulds took a further 10 s, such that the process of creating the specimens was complete within 60 s.

GICs were packed, using a stainless steel dental spatula, into stainless steel moulds with dimensions dependent on the purpose of the specific specimen. Dimensions were: 6 mm height and 4 mm diameter for compressive strength determination; 4 mm height and 6 mm diameter for measurement of diametral tensile strength, release of soluble CHX, and antimicrobial testing; 2 mm height and 15 mm diameter for determination of flexural strength. The moulds were lined with a thin layer of petroleum jelly to aid removal of the set cement. Immediately after packing, the moulds were placed between two sheets of acetate and a 2 kg weight placed on top of the specimens on a flat surface to ensure even distribution of the cement. After 5 min the specimens, except those for biaxial flexural strength testing, were ground on the two flat surfaces using a P120 grit SiC disc and were then placed into small, sealed plastic vessels containing wet tissue paper packed into the lid to achieve 100% humidity without direct contact with water. Specimens were stored at 37 °C for 7 days prior to testing.

### 2.3 Compressive strength (CS) testing

CS was measured by applying a compressive force to the flat surface of the cylindrical specimens (6 mm height, 4 mm diameter) using a universal testing machine (Instron, Buckinghamshire, UK) with cross-head speed of 1 mm/min and recording the load at fracture. Specimen dimensions were measured three times using a digital micrometer. Load at fracture L_F_ was used with diameter D to calculate CS according to the relationship CS = 4L/πD^2^. Specimens were examined after fracture for evidence of flaws on the internal or external surfaces and flawed specimens were rejected. n = 24 specimens per group were used. Data were analysed using a one-way ANOVA followed by a Tukey Honestly Significant Difference post-hoc test at *α* = 0.05.

### 2.4 Diametral tensile strength (DTS) testing

DTS was measured by applying a compressive force to the curved sides of the cylindrical specimen (4 mm height, 6 mm diameter) using a universal testing machine (Instron, Buckinghamshire, UK) with cross-head speed of 1 mm/min and recording the load at fracture. Specimen dimensions were measured three times using a digital micrometer. The load at fracture L_F_ was used in conjunction with the average diameter D and height h of the specimens to calculate DTS according to the relationship DTS = 2L/πDh. Specimens were examined after fracture for evidence of flaws on the internal or external surfaces and data from specimens found to be flawed were rejected. Data were analysed using a one-way ANOVA followed by a Tukey Honestly Significant Difference post-hoc test at *α* = 0.05. As well as freshly prepared specimens (n = 24 per group), the specimens that were subjected to the long-term CHX elution experiments were also tested for DTS to ascertain whether strength deteriorated after long-term exposure to artificial saliva (n = 15 per group).

### 2.5 Biaxial flexural strength (BFS) testing

BFS was measured in a ball-on-ring configuration, by applying force to the flat surfaces of the specimens (15 mm diameter, 2 mm height) using a universal testing machine with cross-head speed of 1 mm/min (Instron, Buckinghamshire, UK) and recording the load at failure. The circular discs were positioned on a 10 mm diameter knife-edge annulus with a thin sheet of rubber between the annulus and the sample. Specimens were centrally loaded with a spherical 4 mm diameter stainless steel ball indentor. BFS (in MPa) was calculated according to the relationship,

$$BFS=\frac{L_{F}}{h^{2}}\left\{\left(1+\upsilon \right)\left[0.484ln\left(\frac{a}{h}\right)+0.52\right]+0.48\right\}$$

where L_F_ = measured load at fracture, a = radius of knife edge support in mm, h = specimen thickness measured at point of fracture in mm and ν = Poisson’s ratio (0.30). n = 24 specimens per group were prepared, and specimens were rejected if there were any visible flaws.

### 2.6 Measurement of elution of soluble CHX

GIC specimens were weighed using a precision balance then placed in individual cuvettes transparent to ultraviolet light (Z637157; Sigma–Aldrich, Gillingham, UK). 1.5 mL artificial saliva was added to each cuvette. The artificial saliva was composed of 0.9 mM CaCl_2_, 0.2 mM MgCl_2_, 4.0 mM KH_2_PO_4_, 30.0 mM KCl, 20.0 mM HEPES buffer, titrated to pH of 6.8 [23]. The cuvettes were sealed with tightly-fitting lids (SEMA2533; VWR, Lutterworth, UK) and then placed onto an orbital shaker (SSM1, Stuart, Staffordshire, UK) at 150 rpm and readings taken initially once a day, and less frequently as CHX release decelerated. Artificial saliva was refreshed at two-week intervals to avoid any decrease in CHX release that could be attributed to saturation of the artificial saliva with respect to CHX salts. Adsorption of light at wavelength 255 nm was measured at regular intervals and calibration standards of 5–50 μM CHX used as references to establish CHX release from the GICs into the artificial saliva [24]. This was converted to μmoles CHX released per unit surface area for each specimen and normalised by subtracting the mean reading for the 0% substitution, correcting for any systematic error due to background readings. n = 15 specimens per group were used. Cuvettes were inspected carefully at each reading and, in the rare event that leakage occurred, the specimen was discarded. CHX elution was measured for 660 days.

### 2.7 Investigation of CHX recharge capacity of GICs

Specimens were prepared as described above and subjected to CHX elution measurements as described in the previous section, with modification to permit the investigation of the capacity for recharge and subsequent release of the CHX. Three sets of specimens (n = 15 each of 0, 1, 2, 5, 10% CHX-HMP for each set) were stored in artificial saliva and CHX release was assessed using UV spectrophotometry as described above. Every 4 weeks the specimens were removed from the artificial saliva and were immersed in one of three “recharge” preparations: deionised water (negative control), 2.2 mM CHX digluconate (“CHX”) and a CHX-HMP suspension containing the equivalent of 2.2 mM CHX (“CHX-HMP”). CHX release of the three groups was assessed to determine whether the specimens periodically exposed to CHX or CHX-HMP had a different subsequent CHX release than those exposed only to water. Readings were converted to μmoles CHX released per unit surface area for each specimen and normalised by subtracting the mean reading for the 0% substitution. Cuvettes were inspected carefully at each reading and, in the rare event that leakage occurred, the specimen was discarded. CHX elution was measured for 315 days.

### 2.8 Assessment of antimicrobial efficacy

*St. mutans* GS-5 [25] was maintained in Brain Heart Infusion broth supplemented with 0.5% (w/v) Yeast Extract (BHY) and incubated for 16 h at 37 °C in a candle jar. *Sc. wiggsiae* C1A_55 [26] was maintained in Tryptic Soy broth supplemented with 0.5% (w/v) Yeast Extract, 25 μg/mL haemin and 5 μg/mL menadione (TSBYHM) and incubated for 5 days at 37 °C anaerobically under N_2_:CO_2_:H_2_ (80:10:10). Bacterial cells were harvested (5000 *g*, 7 min), washed and resuspended in PBS at OD_600_ 1.0 (approximately 2 × 10^8^ cells/mL). Adjusted cell suspensions (100 μL) were spread evenly over BHY agar plates (*St. mutans*) or fastidious anaerobe agar (FAA) plates (*Sc. wiggsiae*), before placing GIC specimens (0 and 2% CHX-HMP) (6 mm diameter, 4 mm height) onto the agar. These lawn plates were then incubated as before for 16 h (*St. mutans*) or 5 days (*Sc. wiggsiae*), before zones of inhibition around the GIC specimens were measured.

## 3 Results

### 3.1 Characterisation of CHX-HMP filler particles

**Fig. 1:**
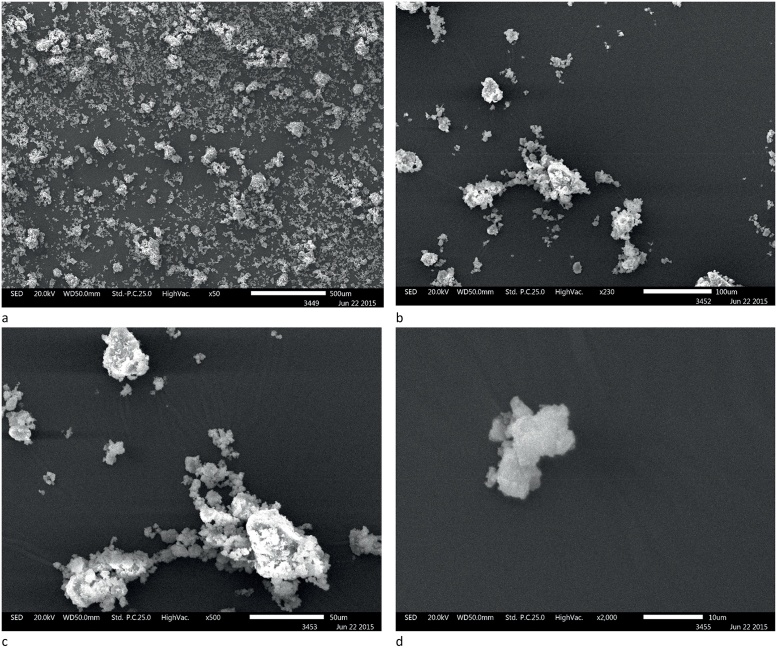
Scanning electron micrographs showing ball-milled CHX-HMP filler particles. Scale bars are a: 500 μm; b: 100 μm; c: 50 μm; d: 10 μm. The particles are polydisperse, with typical sizes ranging from 1 to 20 μm and some aggregates as large as 50–150 μm.

SEM images of the milled CHX-HMP particles are shown in Fig. 1. The milled particles were polydisperse, with the most common sizes in the 1–20 μm range and some aggregates as large as 50–150 μm. The specific surface area of the powder as determined using BET was 1.55 m^2^ g^−1^ and no micropore volume could be measured indicating a very low porosity. Elemental microanalysis indicated that the composition of the precipitate was 20% N, 40% C, 5.3% H and 8.6% P, which corresponds most closely to a composition of 3 CHX to 1 HMP (theoretical values 21% N, 40% C, 4.9% H, 9.3% P). Given the relative molecular masses of CHX and HMP this indicates that the CHX-HMP solid is approximately 76% CHX by mass.

### 3.2 Compressive strength of CHX-HMP functionalised cements

**Fig. 2:**
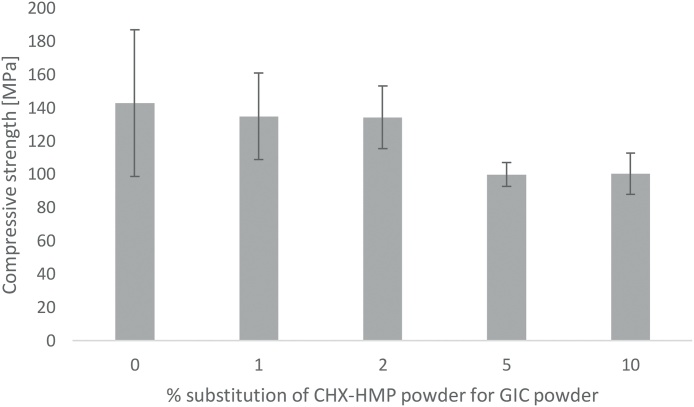
Compressive strength of GIC specimens as a function of CHX-HMP substitution. Error bars represent standard deviations. There was no statistically significant difference between CS of control, 1% or 2% CHX-HMP specimens; 5% and 10% CHX-HMP had significantly lower CS than control, 1% and 2% specimens.

CS data are shown in Fig. 2 with error bars representing standard deviations. Rejection of specimens with internal voids, imperfections or non-linear force-distance curves resulted in final n per specimen group of 17–22 (mean n = 18.6; initial n = 24). According to the ANOVA and Tukey HSD test, there was no statistically significant difference between CS of control, 1% or 2% CHX-HMP specimens; 5% and 10% CHX-HMP had significantly lower CS; that is, substitutions up to and including 2% had no impact of CS compared with control specimens, whereas 5% or more CHX-HMP had a negative impact on CS.

### 3.3 Diametral tensile strength of CHX-HMP functionalised cements

**Fig. 3:**
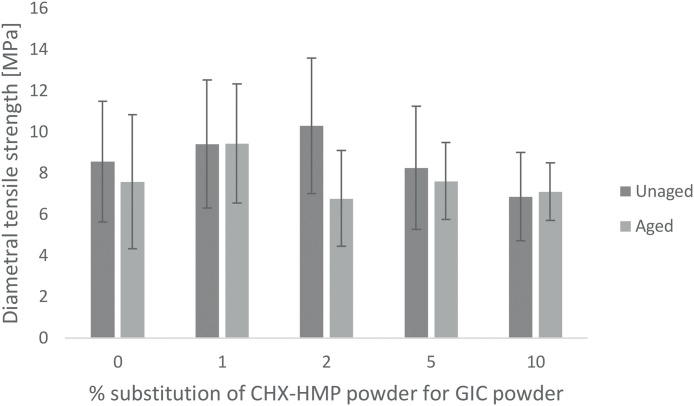
Diametral tensile strength of GIC specimens as a function of CHX-HMP substitution. Error bars represent standard deviations. Dark grey: freshly prepared specimens after storage for 7 days; light grey: aged specimens after 660 days in artificial saliva. The only statistically significant differences were between unaged 2% and 10% CHX-HMP specimens, where 10% CHX-HMP had lower DTS than 2%, and between unaged 2% and aged 2% CHX-HMP specimens, where the aged material had lower DTS than the unaged material.

DTS data are shown in Fig. 3 with error bars representing standard deviations. For specimens tested without aging, rejection of specimens with internal voids, imperfections or non-linear force-distance curves resulted in final n per specimen group of 19–24 (mean n = 21.2; initial n = 24). For specimens tested after immersion in artificial saliva for 660 days, rejection of specimens with internal voids, imperfections or non-linear force-distance curves resulted in final n per specimen group of 8–14 (mean n = 11.2; initial n = 15).

According to the ANOVA and Tukey HSD test, the only statistically significant differences in these data were between unaged 2% and 10% CHX-HMP specimens, where 10% CHX-HMP had lower DTS than 2%, and between unaged 2% and aged 2% CHX-HMP specimens, where the aged material had lower DTS than the unaged material. That is, for unaged specimens substitutions up to and including 10% CHX-HMP had no impact on DTS compared with control specimens, and after aging one specimen group exhibited a reduction in DTS but there were no differences among the groups of specimens comparing all aged specimens.

### 3.4 Biaxial flexural strength (BFS) of CHX-HMP functionalised cements

**Fig. 4:**
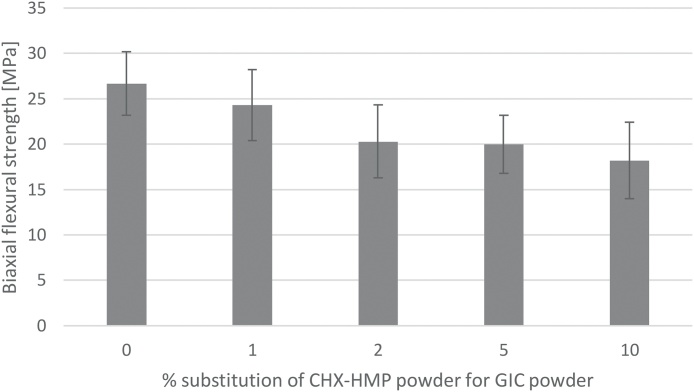
Biaxial flexural strength of GIC specimens as a function of CHX-HMP substitution. Error bars represent standard deviations. The control and 1% CHX-HMP formed one statistically homogeneous group, whereas the others (2, 5 and 10% CHX-HMP) formed a second group with significantly lower BFS than the control and 1% CHX-HMP specimens.

BFS data are shown in Fig. 4 with error bars representing standard deviations. Rejection of specimens with imperfections resulted in final n per specimen group of 22–24 (mean n = 23.0; initial n = 24). According to the ANOVA and Tukey HSD test, the 1% CHX-HMP was indistinguishable from the control group, whereas the others (2, 5 and 10% CHX-HMP) had lower BFS than the control group.

### 3.5 Elution of soluble CHX from functionalised cements

**Fig. 5:**
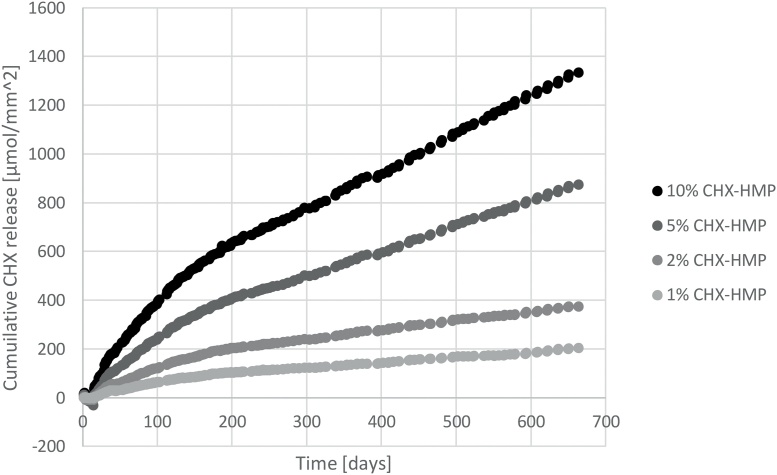
Cumulative CHX release from GIC specimens as a function of CHX-HMP substitution, normalised to control specimens (no CHX-HMP). The elution medium was changed frequently to ensure that at no time the degree of saturation approached the limit of solubility of the CHX HMP salt, ensuring sink conditions throughout.

Elution of soluble CHX from CHX-HMP doped GICs is shown in Fig. 5. A dose-dependent CHX release was seen, with the total CHX released being related, although not directly proportional, to the total CHX content of the material. The CHX release had a smooth profile and did not accelerate and decelerate over the period investigated, indicating that the frequent changing of elution medium ensured that saturation was not a factor in controlling the rate of CHX release.

### 3.6 Investigation of CHX recharge capacity of GICs

**Fig. 6:**
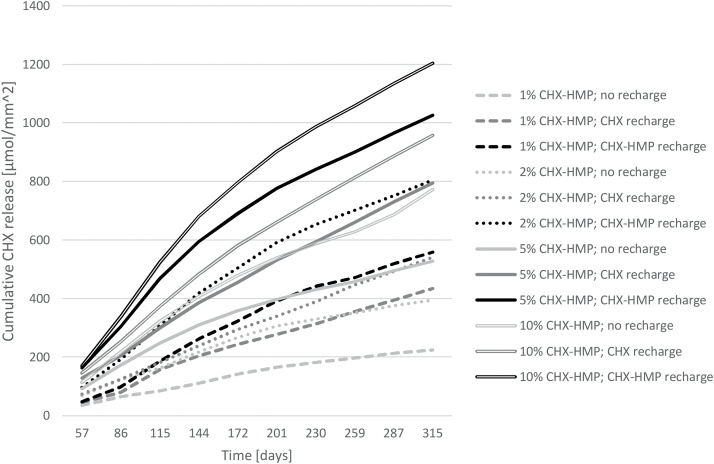
Cumulative CHX release from GIC specimens as a function of CHX-HMP substitution and recharge medium, normalised to control specimens. Specimens recharged with 2.2 mM CHX solution released on average 50% more CHX than negative controls (exposed to DIW). Specimens recharged with CHX-HMP suspension at the same equivalent total CHX concentration released on average 33% more CHX than those recharged with CHX solution and on average 100% more CHX than negative controls.

Elution of soluble CHX from CHX-HMP doped GICs is shown in Fig. 6. Specimens recharged with the 2.2 mM CHX solution released more CHX than negative controls exposed to DIW; on average 50% more CHX was released by the CHX recharged specimens than the controls over the 315 day period. Specimens recharged with CHX-HMP suspension at the same equivalent total CHX concentration released more CHX again than the CHX solution recharged specimens; on average over the 315 period specimens recharged with CHX-HMP released 33% more CHX than those recharged with CHX solution and 100% more CHX than negative controls.

### 3.7 Antimicrobial efficacy of functionalised cements

Control GICs without CHX-HMP showed no remote or contact inhibition of microbial growth with either *St. mutans* or *Sc. wiggsiae*. 2% CHX-HMP GICs resulted in both contact and remote microbial inhibition of *St. mutans*, with an average 11.9 mm zone of inhibition (5.7 mm as measured from the edge of the GIC specimen). 2% CHX-HMP GICs inhibited growth of *Sc. wiggsiae* but did not result in remote inhibition (*i.e*. the inhibition of microbial growth was restricted to the area of agar in direct contact with the specimen).

## 4 Discussion

Incorporation of dry, milled CHX-HMP particles into a commercial GIC resulted in the release of aqueous CHX that was still ongoing at 660 days for all specimen groups. There was a dose response, with higher CHX-HMP dopings releasing more CHX, although this was non-linear, as the total release at 660 days for 2, 5 and 10% CHX-HMP was 1.8x, 4.3x and 6.5x that of the 1% CHX-HMP material respectively. The rate of CHX release decelerated after approximately 150 days, and from that time was close to constant until the conclusion of the study.

The kinetics of CHX release are very different from those observed in other CHX-doped GICs, where there is a rapid decay of CHX release after the first few days of measurement. The fundamental difference is that most of the studies described above utilise CHX digluconate and diacetate which, owing to their solubility, dissipate rapidly in contact with aqueous solutions. CHX-HMP has a much lower solubility than CHX digluconate or diacetate and the rate of CHX release is governed by kinetic factors (agitation of the surrounding medium) and thermodynamic factors (degree of undersaturation of the surrounding medium, which were effectively sink conditions in this study as evidenced by the smooth profile where the 14 day medium changes were not detectable by an acceleration in CHX release). Thus the release profile is not characterised by the “burst” release that has been observed in all studies of CHX digluconate and CHX diacetate GICs.

Utilising the elemental analysis data it can be concluded that the CHX-HMP solid is ∼72% CHX, and this enables calculation of the total CHX contained within the GIC specimens. Given a specimen mass of 202 mg taken from the mean of 152 specimens, this equates to a total content of CHX in the specimens as 1%: 2.9 μmol CHX; 2%: 5.8 μmol CHX; 5%: 14.4 μmol CHX; 10%: 28.7 μmol CHX. At the end of the 660 day period the total CHX released by the specimens was 1%: 0.027 μmol CHX; 2%: 0.049 μmol CHX; 5%: 0.115 μmol CHX; 10%: 0.175 μmol CHX. This indicates that the total CHX release over the 660 day period for all four specimen groups was of the order of 1% of the total CHX contained within the material. It is likely that the CHX that was released was primarily from the outer region of the specimen, where ion exchange with the artificial saliva would most readily take place. It does indicate that the CHX was far from depleted at the endpoint of the study and that continued CHX release would have been likely for a substantially longer time even without recharge. Furthermore, the volume of saliva and the perpetual undersaturation reflects the conditions at the surface of the GIC that would be exposed in the oral environment, and not that interface which would be of most clinical interest, that is, the interface between GIC and tooth tissue. At this location the fluid flow would be very much less and therefore the CHX concentration would likely reach a saturation that would decelerate any further CHX release owing to the common ion effect. To the extent that these observations can be used to infer clinical behaviour it is likely that the duration of CHX release in these experiments underestimates the duration that would be observed at the material-tooth interface *in vivo*.

The capacity to recharge CHX-containing GICs has not previously been investigated. The data indicated that when exposed to either CHX digluconate solution with a concentration equivalent to that in widely used oral rinses, or a CHX-HMP suspension with the same total CHX, subsequent CHX release by the GICs was greater. This indicates that the GIC can take up CHX from its environment, and subsequently release it again in a reversible process. The aim of this part of the study was to determine whether the longevity of the CHX release could potentially be extended by periodic exposure to an oral rinse containing CHX. It appears that this could be effective, and furthermore, a rinse composed of a suspension of CHX-HMP is likely to be more effective, leading to greater CHX release than the CHX digluconate solution. However, the data must be interpreted with caution, since while GICs have long been accepted to take up fluoride from their environment, this ability is now recognised to be short-lived, and is lost after the first month of maturation [27]. Therefore, further investigations regarding the recharge of GICs with chlorhexidine will need to be carried out with more mature specimens to determine whether the same limitations apply.

A primitive assessment of antimicrobial efficacy, the zone of inhibition or agar diffusion method was adopted in this study. The method has many limitations but is commonly used as a first attempt to assess antimicrobial materials, including dental restoratives. It is commonly used in other fields to assess antimicrobial susceptibility of particular microbes. It is important to bear in mind, if comparing different antimicrobials, that the remote effect (the “zone of inhibition”) is affected not only by the interaction between the microbe and the antimicrobial, but also by the ease by which the antimicrobial can diffuse through the agar. CHX, being a comparatively large molecule, diffuses through agar less readily than some antiseptics.

The aim of the disc diffusion study was to take the first steps in assessing whether the CHX release from the prototype GICs was sufficient to have an adverse effect on growth of oral cariogenic microbes, whilst it is acknowledged that more sophisticated, multi-species models would be beneficial in future in ascertaining the optimal dose of CHX-HMP for a given clinical indication. In this simple study, it was illustrated that the 2% CHX-HMP doped specimens inhibited the growth of both *St. mutans* and *Sc. wiggsiae*, with a greater effect on the former microbe.

When adding materials such as CHX salts to GICs it is important to consider the effect that the dissolution of the salts may have on mechanical properties, and the higher the concentration of material the greater the likelihood of an adverse effect on properties such as strength. When adding conventional CHX salts digluconate and diacetate, a dose response of an adverse affect on strength is indeed observed, although the threshold for a statistically significant effect varies from study to study and is likely to be affected by a number of factors including sample size and means of incorporating the CHX. Turkun et al. [6] observed that 0.5% CHX diacetate and 0.5, 1.25 and 2.5% CHX CHX had no adverse effect on CS but higher concentrations of CHX diacetate did reduce CS. There was a numerical reduction in DTS for 2.5% CHX diacetate and digluconate of ∼30% but this was not statistically significant; 0.5 or 1.25% had no effect on DTS either. Ahluwalia observed similarly that 1% CHX diacetate had no adverse effect on the CS or DTS of a GIC, but did not investigate higher or lower concentrations [28]; whereas Mittal who utilised concentrations of 1.5 and 3% CHX diacetate observed a ∼30% reduction in CS compared to unmodified GIC [29], while Duque observed no decrease in CS when adding 1.25 and 2.5% CHX digluconate to a GIC [30]. While it is therefore not possible to draw a definitive conclusion as to what concentration of CHX salts has a significant (whether statistical or clinical) effect on strength, a likely explanation of the reduction in CS and DTS observed by some authors with higher concentrations may be explained Marti’s observation that increased concentration of CHX diacetate or digluconate is accompanied by an increase in porosity [10] which is likely to reduce strength.

The higher the substitution of CHX-HMP, the more likely a deleterious effect on the mechanical properties, with substitutions of 5% or greater adversely affecting CS, and substitutions of 2% or above adversely affecting BFS, although none significantly affected DTS. At the higher concentrations of 5 and 10%, the CS was 100 MPa which is the minimum CS stipulated by the relevant ISO standard for restorative cements [31]; lower concentrations brought the strength more comfortably within the acceptable range at 130–150 MPa. The threshold for adverse effects on the mechanical properties of these materials appears therefore to be broadly comparable, or perhaps a little higher, than that for more soluble CHX salts, although it would be interesting to explore how these mechanical properties vary over time as the CHX is released from the various GIC materials.

It is considered encouraging that for the lower dopings of CHX-HMP the effects on the mechanical properties are moderately small; should such materials become commercially viable, it is plausible that other aspects of the formulation could be modified to compensate for this loss of strength. On the other hand, these GIC materials could be considered as prototypes for GIC-based fissure sealants, where absolute strength is of lesser importance and the local delivery of antimicrobial to the fissure areas could be considered beneficial, and as temporary materials designed to stabilise the region before the definitive restoration is placed. However, given the calculations presented above regarding the proportion of CHX released from the specimens, and the reflections on relative fluid flow rate and therefore CHX saturation at the tooth-tissue interface, it is plausible that even the lower concentrations of CHX-HMP used here may still offer sufficient CHX release to operate as long-term restoratives without compromise to mechanical properties.

## 5 Summary

GICs have been developed which are supplemented with fine, milled, CHX-HMP particles. CHX release was sustained for considerably longer and at a more consistent rate than with any previous published studies, and with fewer adverse effects on mechanical properties than many formulations with short lived and front-loaded CHX release. Earlier studies with CHX-HMP prepared in different ways have achieved long term CHX release, for example ongoing release after over 400 days in artificial saliva, but this was at the expense of mechanical properties even using much smaller CHX-HMP dopings. The materials presented in this manuscript may prove beneficial as technologies with the aim of inhibiting secondary caries in mainstream restorative materials, as well as related materials such as temporary restorations and GIC-based fissure sealants.

## Funding

The study was funded by the Medical Research Council as part of a CASE PhD studentship awarded to Candice Bellis. Kemdent were the CASE partner and provided in-kind support but did not take an active role in the data acquisition, interpretation or analysis of data, or the decision as to whether and how to publish the data. PFD was supported by EPSRC under its ACCIS Centre for Doctoral Training grant, EP/G036772/1.
